# Association between adolescent emotional problems and metabolic syndrome: The modifying effect of C-reactive protein gene (*CRP*) polymorphisms

**DOI:** 10.1016/j.bbi.2011.01.019

**Published:** 2011-05

**Authors:** Darya Gaysina, Mary Pierce, Marcus Richards, Matthew Hotopf, Diana Kuh, Rebecca Hardy

**Affiliations:** a MRC Unit for Lifelong Health and Ageing, University College London, London, UK; b King’s College London, Institute of Psychiatry, London, UK

**Keywords:** Inflammation, Obesity, Depression, Anxiety, CRP, Gene

## Abstract

Depression is associated with the development of the metabolic syndrome, and both depression and metabolic syndrome are associated with markers of systemic inflammation, such as C-reactive protein (CRP). We examined associations between affective status in adolescence and adulthood, and the metabolic syndrome at age 53 years in a large representative British birth cohort. We also investigated whether two *CRP* gene polymorphisms (rs1205 and rs3093068) were associated with affective status and the metabolic syndrome, and whether the association between affective status and the metabolic syndrome was modified by these *CRP* polymorphisms. Women, but not men, with emotional problems in adolescence were more likely to have the metabolic syndrome (OR = 1.53, 95% CI: 1.04, 2.26), although this sex difference was not statistically significant (*p* = 0.22). The *CRP* SNPs were not associated with affective status or the metabolic syndrome, but the association of adolescent emotional problems with the metabolic syndrome was stronger in those who were homozygous for the major allele (C) of rs1205 (OR = 1.83, 95% CI: 1.17, 2.86) than in carriers of the T allele (OR = 1.01, 95% CI: 0.66, 1.55) (*p* = 0.05 for gene by affective status interaction). This interaction was stronger when considering adolescent emotional problems as a continuous variable (*p* = 0.003). Adolescent emotional problems play an important role in the development of the metabolic syndrome later in life, particularly in those homozygous for the major allele of *CRP* rs1205. These findings may highlight new ways of identifying people with emotional problems at high risk of developing the metabolic syndrome, which is of great importance for the management of the physical health of these patients.

## Research highlights

► Adolescent emotional problems are a risk factor for metabolic syndrome in mid life. *► CRP* gene modifies the association. ► The association is stronger in *CRP* rs1205 CC homozygotes than in T allele carriers. *► CRP* gene helps identifying depressed people at high risk for metabolic syndrome.

## 1 Introduction

The association between depression and the metabolic syndrome has assumed greater public health importance due to the rapidly increasing prevalence of these disorders during the past two decades (British Nutrition Foundation, 2004; Ford et al., 2002; World Health Organization, 2008). All relevant longitudinal studies suggest a higher incidence of the metabolic syndrome and/or its components (high waist circumference, high triglyceride level, low HDL level, high blood pressure, and high glucose level) among those with depressive symptoms (Raikkonen et al., 2002, 2007; Goldbacher et al., 2009; Pulkki-Raback et al., 2009; Vaccarino et al., 2008; Vanhala et al., 2009; Viinamaki et al., 2009), even if different definitions for the metabolic syndrome were used (Expert Panel on Detection, Evaluation, and Treatment of High Blood Cholesterol in Adults, 2001; Grundy et al., 2004). Much less attention, however, has been paid to the association between anxiety and the metabolic syndrome, with the few existing cross-sectional studies reporting mixed findings (Carroll et al., 2009; Dunbar et al., 2008).

So far, the biological mechanisms underlying the association between depression and anxiety and later metabolic syndrome remain poorly understood. One biological factor receiving increased attention as a potential mechanism for this association is inflammation. C-reactive protein (CRP) is a non-specific marker of systemic inflammation. Its concentration rises as much as 2000-fold during the first 24–48 h after the onset of tissue injury or inflammation. Higher CRP plasma levels have been shown to be associated with both the metabolic syndrome (Devaraj et al., 2009) and depression (Raison et al., 2006; Gimeno et al., 2009; Howren et al., 2009) and anxiety (Bankier et al., 2009; Pitsavos et al., 2006), although some studies failed to confirm these associations (Douglas et al., 2004; O’Donovan et al., 2010; Whooley et al., 2007). Among the possible reasons for these inconsistent findings is the potential confounding of the relationship by factors such as lifestyle and socioeconomic conditions. Genetic studies have the potential to shed light on the role of CRP in the relationship between depression and anxiety and the metabolic syndrome, since genes influencing CRP levels will not be influenced by these potential confounding factors. It has been estimated that the interindividual variability in blood CRP level is 35–52% heritable (Pankow et al., 2001; MacGregor et al., 2004) and certain single nucleotide polymorphisms (SNPs) of the *CRP* gene have been found to strongly influence the blood level of CRP (Kolz et al., 2008; Almeida et al., 2009; Lee et al., 2009). Two *CRP* SNPs, rs3093068 and rs1205, have been associated with variation in CRP level, with the C allele of rs3093068 and the C allele of rs1205 being associated with higher level of plasma CRP (Kolz et al., 2008; Halder et al., 2010).

To date, only a few studies have investigated *CRP* variations in relation to the metabolic syndrome, with some providing null results (Evrim et al., 2009; Timpson et al., 2005), and the most recent reporting a significant association (Hsu et al., 2010). Similarly, only two studies have evaluated associations between *CRP* polymorphisms and depression: one study reported a significant association between *CRP* rs1205 polymorphism and clinically significant depression in men (Almeida et al., 2009), while another found no effect of three *CRP* polymorphisms or haplotypes on depressive scores (Halder et al., 2010).

To the best of our knowledge, no studies have tested the role of *CRP* polymorphisms in the relationship between affective status and the metabolic syndrome or considered whether the *CRP* gene modifies risk for metabolic syndrome in those who have affective symptoms, even though gene-environment interactions are likely to contribute to a complex trait such as metabolic syndrome. Further, the *CRP* gene has been found to modify the relationship between depressive symptoms and circulating CRP level (Halder et al., 2010) suggesting the possibility of such *CRP* gene by depression interactions in relation to risk of the metabolic syndrome.

In the current study, we hypothesize that the *CRP* gene is an important candidate gene for understanding the affective status–metabolic syndrome association. It may be involved in plausible biological pathways for each of these conditions. Alternatively, the genetic effect may represent an altered predisposition to the metabolic syndrome in those who have affective symptoms. The aim of this study, using data from the British 1946 birth cohort, is to test: (1) whether emotional problems in adolescence and adulthood are associated with the metabolic syndrome in midlife; (2) whether two *CRP* polymorphisms, rs1205 and rs3093068, are associated with the metabolic syndrome and whether they are associated with adolescent emotional problems and adult affective symptoms; (3) whether any association between the *CRP* gene and the metabolic syndrome is mediated through affective status; and (4) whether there is an interaction between affective status and *CRP* genetic variants in relation to risk of the metabolic syndrome.

## 2 Methods

### 2.1 Study participants

The Medical Research Council (MRC) National Survey of Health and Development (NSHD) (also known as the British 1946 birth cohort) initially consisted of a stratified sample of 5362 children born within marriage in England, Scotland and Wales during one week in March 1946. The cohort has been studied on 21 occasions since birth, most recently in 1999 when cohort members were aged 53 years, when sample size was 3035. At age 53 years the responding sample remained reasonably representative of the British born population of the same age (Wadsworth et al., 2006).

### 2.2 Teacher-rated adolescent emotional problems

Assessment of adolescent emotional problems was based on questionnaires completed by teachers when survey members were aged 13 and 15 years, describing personality, behaviour, and mood (Rutter, 1967). These questionnaires have previously been subjected to factor analysis. Items that loaded onto the emotional problems (depression and anxiety) factor were “timid child,” “rather frightened of rough games,” “extremely fearful,” “always tired and washed out,” “usually gloomy and sad,” “avoids attention,” “very anxious,” “unable to make friends,” “diffident about competing,” “frequently daydreams in class,” and “becomes unduly miserable or worried in response to criticism” (Colman et al., 2007; van Os et al., 1997). Cronbach’s alpha was calculated for the scale at both ages 13 and 15, with scores of 0.68 and 0.71, respectively, indicating that the scale was reliable.

Adolescents were considered to have emotional problems if they scored above the 88th percentile on the factor. The 88th percentile was chosen because a prevalence of 12% corresponds to reported prevalence of adolescent affective disorder (Office of Applied Studies, 2005; Costello et al., 1996). It has previously been shown that adolescents who had emotional problems at both ages 13 and 15 years had a significantly higher risk of mental disorder at ages 36, 43, or 53 years. They were also more likely than adolescents without emotional problems to have self-reported “nervous trouble” and to have been treated for psychiatric disorder during adulthood (Colman et al., 2007). We decided to use the binary variable for adolescent emotional problems in analyses in order to make results comparable with analyses using the measure of affective symptoms at age 36 years, and because of our interest in the effect of the more clinical symptoms. We also repeated all analyses with the continuous measure of adolescent emotional problems.

### 2.3 Adult affective symptoms

Frequency and severity of affective symptoms (depression and anxiety) were assessed in adulthood, using the Present State Examination (PSE) (Wing et al., 1974) at age 36 years. A shortened version of the PSE was administered by trained nurses to obtain standardised interview ratings of low mood, anxiety, and phobia symptoms in reference to one month prior to the interview. A computer-generated, previously validated categorical variable was created from this 48-item diagnostic assessment through an index of definition (ID) where 5 or higher was taken as evidence of affective symptoms (6.2% of the population).

### 2.4 Metabolic syndrome

During the interview at age 53 years, the research nurses measured waist circumference, blood pressure and took non-fasting blood samples from which lipids and HbA1c were obtained. We defined the metabolic syndrome and its components using cut-points recommended by ATPIII8 (2001); we modified this definition to include HbA1c instead of fasting plasma glucose, data for which were unavailable (see Langenberg et al., 2006). HbA1c is a reliable estimate of usual glycaemia over the preceding 6–12 weeks and has been shown to predict mortality continuously across the entire population distribution in people without diabetes (Khaw et al., 2001, 2004). Participants were classified as having the metabolic syndrome if they met any three of the following criteria: waist circumference >102 cm for men or >88 cm for women, triglyceride level ⩾1.7 mmol/L (150 mg/dL), HDL cholesterol level <1.036 mmol/L for men or <1.295 mmol/L for women, blood pressure level ⩾130/85 mm Hg, or HbA1c level in the top gender-specific quarter of the distribution (>5.8% among both men and women). Participants taking British National Formulary (BNF)-classified antihypertensive medications (diuretics, beta blockers, drugs affecting the renin–angiotensin system, and calcium-channel blockers) or BNF-classified diabetes medications were classified as meeting high blood pressure and HbA1c criteria, respectively. Additional analyses using the top quintile of HbA1c (above 5.9%), rather than quartile, as the cut-off were carried out to assess the sensitivity of our findings to the choice of cut-point.

### 2.5 SNP selection and genotyping

We investigated two single nucleotide polymorphisms (SNP) of the *CRP* gene, rs1205 and rs3093068. These SNPs have been shown to be associated with plasma CRP concentration (Halder et al., 2010; Kolz et al., 2008). DNA was extracted and purified from whole blood using the Puregene DNA Isolation Kit (Flowgen, Leicestershire, UK) according to the manufacturer’s protocol. The SNPs were typed by Source Bioscience PLC using the Applied Biosystems (Foster City, CA) SNPlex technology which is a based on an Oligonucleotide Ligation Assay combined with multiplex PCR amplification and capillary electrophoresis. Genotyping was performed using an ABI 3730xl DNA Analyser and ABI GeneMapper v4.0 software. The integrity of the genotyping was checked by genotyping frequency, concordance of duplicates and Hardy–Weinberg equilibrium (HWE). The call rates for the SNPs was >99%, with >95% concordance between duplicate samples. There was no evidence of deviation from HWE in the total sample or in the investigated sub-groups (*p* > 0.05).

### 2.6 Statistical analysis

We used logistic regression models to assess associations between adolescent emotional problems (at age 13–15 years), and between adult affective symptoms (at age 36 years) and the metabolic syndrome and its components (at age 53 years). In addition to the main analyses, sensitivity analyses were carried out to investigate the possibility that any relationship observed may be influenced by reverse causality. Given that the causal direction of the association between affective symptoms and the metabolic syndrome remains unknown, it is possible that any observed relationship between affective symptoms and the metabolic syndrome is due to a pre-existing metabolic syndrome resulting in affective symptoms. Since information to allow ascertainment of the metabolic syndrome before age 53 years was not available, individuals most likely to have early onset metabolic syndrome were excluded from these sensitivity analyses to ensure that occurrence of affective symptoms preceded the onset of the metabolic syndrome. We excluded those who were overweight at age 15 years when considering adolescent emotional problems, and those who had diabetes or BMI ⩾ 30 kg/m^2^ at age 36 years when considering adult affective symptoms.

We then fitted a model with metabolic syndrome as the outcome with both adolescent emotional problems and adult affective symptoms as explanatory variables. All models were adjusted for sex. Tests were then carried out to assess whether the associations were the same in men and women by adding a sex by affective status interaction term in addition to the main effects of sex and affective status. In addition, analyses were carried out separately for men and women.

Pairwise linkage disequilibrium (LD) was ascertained using the Haploview 4.0 (Barrett et al., 2005). Linkage disequilibrium between the two investigated SNPs was low (*r*^2^ = 0.03). We therefore chose to test separately the association between the phenotypes of interest and each of the SNPs. Associations between each *CRP* gene polymorphism and the metabolic syndrome at age 53 years, as well as between each *CRP* gene polymorphisms and emotional problems in adolescence and affective symptoms in adulthood, were tested using five different genetic models (allelic, genotype, dominant/recessive, recessive/dominant, and additive). Each polymorphism was then added separately to the logistic regression model investigating the association between affective symptoms and the metabolic syndrome to assess whether either attenuated the relationship. If an association between any polymorphism of *CRP* gene and the metabolic syndrome was observed, we then assessed whether it was mediated by adolescent emotional problems or adult affective symptoms (MacKinnon et al., 2007).

To test for an interaction between affective status and genotype, a multiple logistic regression model was fitted with the metabolic syndrome as the outcome and genotype (based on the dominant/recessive genotype model), affective status and their interaction term as predictor variables and sex as a confounding variable. Data were managed and analysed with the statistical package Stata release 10.0 (StataCorp, College Station, TX, USA).

## 3 Results

### 3.1 Descriptive statistics

Every survey member with information on affective status who had at least one clinical measure of the metabolic syndrome at age 53 years was included in the descriptive analysis (*n* = 2658 with adolescent emotional problems and 2676 with adult affective symptoms). Table 1 shows the results of the descriptive analyses for the metabolic syndrome components by adolescent and adult affective status. Those with information on all clinical measures were available for the analysis of the metabolic syndrome: there were 2078 men and women with full information on the metabolic syndrome status among those with information on adolescent emotional problems, and 2105 with full information on the metabolic syndrome status among those with information on adult affective symptoms at age 36 years. The frequency of adult affective symptoms did not differ between those with and those without the information on the metabolic syndrome at age 53 (*p* = 0.73). Those with metabolic syndrome information had slightly lower levels of adolescent emotional problems than those without (*p* = 0.06).

The genotype distributions for the *CRP* SNPs were similar in men and women. For rs1205, the distribution was: 44.4% (CC), 45.1% (CT), 10.5% (TT) in men (*N* = 1240), and 44.5% (CC), 45.8% (CT), 9.6% (TT) in women (*N* = 1237) (*p* for sex difference in genotypes = 0.73, alleles = 0.67). For rs3093068, the genotype distribution was: 88.1% (CC), 11.6% (CG), and 0.3% (GG) in men (*N* = 1239), and 89.7% (CC), 10.1% (CG), and 0.2% (GG) in women (*N* = 1231) (*p* for sex difference in genotypes = 0.47, alleles = 0.24).

### 3.2 Association between affective status and the metabolic syndrome

In the total sample, the prevalence of the metabolic syndrome was higher in those with adolescent emotional problems (32.9%) and in those with adult affective symptoms (34.1%) than in those without (28.2% and 28.4%, for adolescence and adulthood, respectively). The differences in prevalence were similar in both cases, with confidence intervals just including the null value of zero (Table 1).

In women, adolescent emotional problems were associated with higher odds of the metabolic syndrome (23.2% in those without emotional problems versus 31.7% in those with emotional problems: OR = 1.53, 95% CI: 1.04, 2.26) (top half of Table 2). There was a suggestion that the association may be weaker in men than women although the test for interaction did not reach conventional significance levels (*p* = 0.22, OR for interaction = 1.44, 95% CI: 0.80, 2.59). Using the continuous measure of adolescent emotional problems the same association was observed in women (OR = 1.32 per one score increase, 95% CI: 1.00, 1.75), but not in men (OR = 1.12, 95% CI: 0.87, 1.46). Similarly, a higher risk of the metabolic syndrome was observed in women with affective symptoms at age 36 years than in those without (23.9% without affective symptoms versus 32.6% with affective symptoms: OR = 1.54, 95% CI: 0.97, 2.46) (bottom half of Table 2). However, there was no evidence of a statistical difference in the association between men and women (*p* for sex interaction = 0.53; OR = 1.29, 95% CI: 0.59, 2.83).

Adolescent emotional problems were associated with high HbA1c level in the total sample (OR = 1.46, 95% CI: 1.11, 1.93) (top half of Table 1). Adult affective symptoms showed the strongest relationship with high triglyceride levels (bottom half of Table 1). For women, the associations between adolescent emotional problems and all components of the metabolic syndrome, except HDL cholesterol, are in the same direction (Table 2). Similar consistency in the direction of most associations is also seen for adult affective symptoms. For men, the direction and size of associations are varied. In men, childhood emotional problems are only associated with raised Hba1c, and adult affective problems are strongly associated with hypertension (OR = 2.62, 95% CI 1.03–6.69). This association was not observed in women (OR = 1.01, 95% CI 0.68–1.51) and there was evidence of a sex difference in this relationship (*p* for sex interaction = 0.07; OR = 0.39, 95% CI: 0.14, 1.07) (bottom half of Table 2).

Analyses including both adolescent emotional problems and adult affective symptoms as predictors of metabolic syndrome in women result in slight decreases in both ORs when compared with the unadjusted estimates. Confidence intervals for both variables, however, now include 1 suggesting that these measures may not operate independently (adolescent emotional problems: OR = 1.46, 95% CI: 0.97, 2.18; adult affective symptoms: OR = 1.52, 95% CI: 0.93, 2.47).

Sensitivity analysis excluding women with BMI ⩾ 25 kg/m^2^ at age 15 years produced a slightly stronger association between adolescent emotional problems and the metabolic syndrome (OR = 1.66, 95% CI: 1.07, 2.58). The findings among men (OR = 0.96, 95% CI: 0.58, 1.57) and in a total group (OR = 1.29, 95% CI: 0.93, 1.80) remained unchanged. Thus, the association between adolescent emotional problems and metabolic syndrome was stronger in women than in men (*p* for sex interaction = 0.10; OR = 1.73; 95% CI: 0.89, 3.36).

When we excluded those with type 2 diabetes diagnosed before age 36 years or obesity at this age, the association between adult affective symptoms and the metabolic syndrome remained unchanged in women (OR = 1.54, 95% CI: 0.93, 4.33), as it did in men (OR = 1.25, 95% CI: 0.64, 2.43), and in a total group (OR = 1.43, 95% CI: 0.95, 2.13). The association between adult affective status and metabolic syndrome did not differ between men and women (*p* for sex interaction = 0.62; OR = 1.24; 95% CI: 0.54, 2.85).

Sensitivity analyses using the top quintile (HbA1c above 5.9%), rather than the top quartile, as the cut-off for defining the risk group revealed that results were essentially unchanged.

### 3.3 CRP gene, affective status and the metabolic syndrome

As there were no sex differences in *CRP* genotype or allele distribution, and sex by genotype interactions for both affective status and metabolic syndrome were not significant, the analysis of genetic associations are presented in the sample with men and women combined. We found no associations between *CRP* polymorphisms and the metabolic syndrome or between *CRP* polymorphisms and affective status in adolescence or adulthood for both allele and genotype models (Table 3). Similarly, no associations were found between genotypes and the continuous measure of adolescent emotional problems (data not shown).

Inclusion of affective status in the model of the association between *CRP* polymorphisms and metabolic syndrome did not influence the non-significant relationship. These findings indicate that the relationship between CRP polymorphisms and the metabolic syndrome cannot be mediated through affective status.

**Fig. 1:**
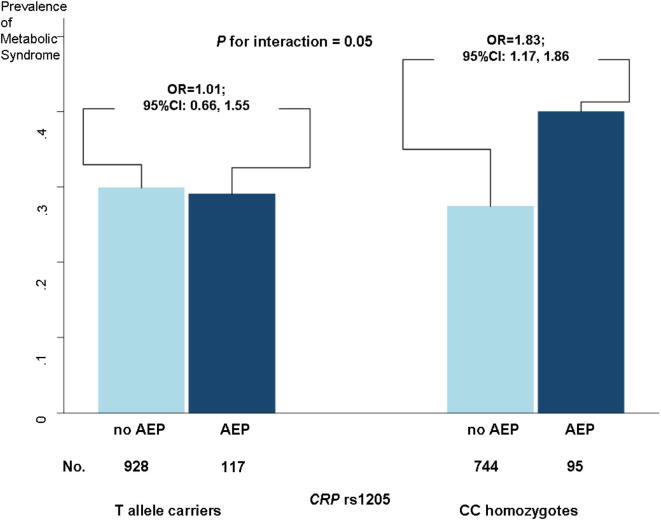
The effect of adolescent emotional problems on the risk of the metabolic syndrome among CC homozygotes and T allele carriers of the *CRP* rs1205 polymorphism. Odds ratios are derived by logistic regression and adjusted for sex. No AEP = no adolescent emotional problems; AEP = adolescent emotional problems.

To test the interaction between the *CRP* polymorphisms and affective status on risk of the metabolic syndrome, we grouped the genotypes into binary variables according to the existing literature on the effect of these polymorphisms on plasma CRP concentration (Kolz et al., 2008). For rs1205, CT and TT genotypes were combined (dominant model for minor T allele) representing a group with lower plasma CRP level. For rs3093068, CG and GG genotypes were combined (dominant model for minor G allele) representing a group with higher CRP level. There was a significant interaction between *CRP* rs1205 genotype and affective status in adolescence (*p* = 0.05). Stratifying the study population by *CRP* rs1205 genotype group showed that adolescent emotional problems were associated with the metabolic syndrome among CC homozygotes (OR = 1.83, 95% CI: 1.17, 1.86) with very similar differences in predicted probabilities for men (14.2%, 95% CI: 3.3, 25.1) and for women (12.6%, 95% CI: 2.6, 22.6), but not among T allele carriers (OR = 1.01, 95% CI: 0.66, 1.55; differences in predicted probabilities = 0.3%, 95% CI: −9.6, 10.1 for men, and = 0.02%, 95% CI: −7.6, 8.0 for women). These results are presented in Fig. 1. A similar, but stronger, interaction was found when using the continuous measure of adolescent emotional problems (*p* for interaction = 0.003): increased risk for metabolic syndrome was associated with increased adolescent emotional problems in those homozygous for the C allele (OR = 1.75 per one score increase, 95% CI: 1.28, 2.41), but not for T allele carriers (OR = 0.95 per one score increase, 95% CI: 0.72, 1.25).

There was no evidence of interactions between adolescent emotional problems and *CRP* rs3093068 or between adult affective symptoms and either of the *CRP* polymorphisms.

## 4 Discussion

This is the first longitudinal population-based study to investigate potential genetic mechanisms underlying the associations between adolescent and adult affective status and the metabolic syndrome. Our findings provide evidence of an association between adolescent affective status and the metabolic syndrome in women but not in men, although this sex difference was not statistically significant. *CRP* gene variants were not associated with the metabolic syndrome, but the association between adolescent emotional problems and later metabolic syndrome was modified by *CRP* rs1205. Adolescent emotional problems were strongly related to the metabolic syndrome among CC homozygotes, but not among T allele carriers of the *CRP* rs1205 polymorphism.

Several limitations should be taken into account when interpreting the present findings. The metabolic syndrome ascertainment was not possible in adolescence or early adulthood, thus we can not be certain of the direction of causality of the association between affective symptoms and metabolic syndrome. However, the sensitivity analyses excluding those most likely to have metabolic syndrome at earlier ages, that is those overweight in adolescence and those who had type 2 diabetes or high waist circumference at age 36 years, did not significantly alter the strength of the associations. Moreover, there was little obesity (BMI > 30 kg/m^2^) in childhood or adolescence in this cohort (7% for girls and 3% for boys at age 15) compared with the modern population aged 2–15 (15% of girls and 17% of boys), and the prevalence of the metabolic syndrome was likely to be considerably lower than in current adolescents (The Health Survey for England, 2008).

We used teacher’s ratings of adolescent mental health, which we acknowledge may differ from self-reported adolescent internalizing symptoms (Auger, 2004). The validity of the adolescent measure of emotional problems derived from teacher questionnaires is, however, supported by several lines of evidence. First, it has been shown that teachers may identify psychiatric problems in children more accurately than parents or the children themselves (Kresanov et al., 1998). Second, teacher-rated psychiatric problems more accurately predict future psychiatric disorder than psychiatric problems based on parent or child ratings (Sourander et al., 2004). In the 1946 birth cohort, a strong association has been observed between the teacher rating measures and adult mental health and later use of mental health services and has previously been used to define adolescent internalizing disorder (Colman et al., 2007).

Although a CRP plasma level measure was not available in the cohort, several previous studies have reported that rs1205 and rs3093068 significantly influence the CRP plasma level (Halder et al., 2010; Kolz et al., 2008). SNP rs3093068 is in LD with other *CRP* SNP rs3093062, which lies within an evolutionarily conserved region of the *CRP* promoter and are predicted to alter a transcription factor E box binding element (Carlson et al., 2005; Szalai et al., 2005). Furthermore, *in vitro* assays have demonstrated the functional significance of rs3093062 in the promoter region of *CRP* (Carlson et al., 2005; Szalai et al., 2005). The functional significance of rs1205 is more difficult to understand. SNP rs1205 is located distal to the 3′ untranslated region of *CRP* and in the MLT1K repeat (Crawford et al., 2006). It is likely that there are other polymorphic variants of functional importance within the gene. A better coverage with tag SNPs would require in order capturing other possible functional variants. However, it has been shown that there is extremely strong LD over and upstream of the CRP gene where the both investigated SNPs located (Eiriksdottir et al., 2009; Hage and Szalai, 2007). So it is unlikely that haplotypes would add beyond the effect of the single SNPs within these regions.

We have not formally tested for population stratification; however the 1946 birth cohort was formed before the beginning of large-scale immigration from Commonwealth countries and is thus entirely of white Caucasians.

Loss to follow-up and missing data are unavoidable in long running birth cohort studies such as the NSHD. At age 53 years the NSHD remains, in most respects, representative of the British born population of the same age (Wadsworth et al., 2006). There were only minor differences in level of adolescent affective symptoms and no difference in adult affective symptoms between those included and those excluded from our analyses. To weaken the observed association between adolescent emotional problems and risk of the metabolic syndrome, “missingness” would have to be more common for people with an absence of adolescent emotional problems and higher risk for metabolic syndrome. We cannot see any reason why this should be the case.

Our study has several methodological strengths. Our study has a 40 year follow-up from initial measurement of affective status at age 13 years, the longest follow-up for a longitudinal study of depression and the metabolic syndrome. It therefore provides the opportunity to explore the effect of age of onset of the mental health problems on the metabolic syndrome later in life. Moreover, our study has the biggest sample size (*N* ⩾ 2221) in comparison with the previous epidemiological longitudinal studies of the association between affective symptoms and metabolic syndrome, where the maximum number of participants is approximately 1300 participants (Vanhala et al., 2009). Despite a large number of studies linking depression and anxiety to elevated CRP level (Bankier et al., 2009; Howren et al., 2009; Pitsavos et al., 2006), so far there has been only two studies investigating *CRP* genetic variants in depression (Almeida et al., 2009; Halder et al., 2010), and none investigating these *CRP* variants and the metabolic syndrome in those with affective symptoms.

The results of the present analyses are consistent with previous longitudinal studies reporting that depression (Raikkonen et al., 2002, 2007; Vaccarino et al., 2008; Vanhala et al., 2009; Goldbacher et al., 2009; Pulkki-Raback et al., 2009; Viinamaki et al., 2009) is a risk factor for the development of the metabolic syndrome. Four of these studies included women only (Raikkonen et al., 2002, 2007; Vaccarino et al., 2008; Goldbacher et al., 2009), and three others included both sexes (Viinamaki et al., 2009; Pulkki-Raback et al., 2009; Vanhala et al., 2009). The three studies including men and women observed sex differences in the association between depression and the metabolic syndrome. Consistent with our findings, two studies reported an association in women but not in men (Vanhala et al., 2009; Pulkki-Raback et al., 2009), while one study found an association in men but not in women (Viinamaki et al., 2009). In our study significant gender differences were revealed for the association with one metabolic component – hypertension; an association between higher affective symptoms and hypertension at age 53 years was observed in men, but not women.

There are several unique features of the metabolic syndrome in women (Scuteri et al., 2009), and depression is twice as high in women as in men, with the rate beginning to rise rapidly in adolescence. A large number of studies suggest that adolescent emotional problems in girls, but not in boys, lead to significant weight gain and/or obesity during the life course (Liem et al., 2008; Blaine, 2008). Depressed women could be at increased risk for the metabolic syndrome through effects on adiposity, lipid metabolism and inflammation (Schneider et al., 2006). These associations could be due to poor dietary and exercise habits in depressed adolescent girls (Strine et al., 2008; Fulkerson et al., 2004) and the tracking of these poor health behaviours into adulthood. When comparing the prevalence of metabolic syndrome in men and women, it is women without adolescence emotional problems who are protected against the metabolic syndrome in later in life compared with all men and women with emotional problems. This might suggest that it is better education and more healthy lifestyles across adult life in these women that is protective. A study of longitudinal paths to the metabolic syndrome demonstrated that high blood pressure was not a risk factor for the future development of the metabolic syndrome (Scuteri et al., 2009). This could explain why we observed an association of affective symptoms with hypertension, but not with the metabolic syndrome, in men. On the other hand, the previous studies could have had insufficient power to detect an association between depression and metabolic syndrome in men since depression is less common in men than in women.

We used a combined trait of depression and anxiety since there is a strong comorbidity between mood and anxiety disorders (Lewinsohn et al., 1997; Kessler et al., 2005; Essau, 2003), and since there is evidence for an association between both disorders and the metabolic syndrome. The multidimensional nature of affective disorders may influence its relationship with the metabolic syndrome (Watson, 2009). It is likely that some dimensions such as fatigue may be strongly associated with this syndrome (Maloney et al., 2010), while others (e.g., thoughts of worthlessness) may not. In this case, using the complex trait of affective disorders may result in a weaker relationship. Future research is required to assess which dimensions are most strongly associated with the metabolic syndrome.

Emerging laboratory and epidemiological data suggest that CRP is an important plausible factor for insulin resistance, adiposity and other features of the metabolic syndrome (Devaraj et al., 2009). For instance, two recent studies have provided the evidence that CRP impairs insulin signalling (D’Alessandris et al., 2007; Xu et al., 2007). Additionally, it has been demonstrated both *in vitro* and *in vivo* that CRP impairs endothelial vasoreactivity, and hence could increase the risk for hypertension (Guan et al., 2009; Singh et al., 2007). However, findings of the genetic studies investigating the role of *CRP* in inflammatory diseases are not consistent. One study of the *CRP* gene in metabolic syndrome showed no association (Timpson et al., 2005), while the most recent study reported a significant association (Hsu et al., 2010). The results of two studies of *CRP* and obesity using a similar Mendelian randomization approach also contradict each other, with one reporting CRP is causally and positively related to BMI in women (Bochud et al., 2009), while another arguing that there is no evidence that higher CRP level causes greater adiposity (Welsh et al., 2010). A Mendelian randomization approach does not take into account possible gene-environmental interactions, which are most likely to contribute to such a complex trait as metabolic syndrome.

The current study provides novel evidence for a depression-by-*CRP* gene interaction effect on the metabolic syndrome. Similar to the previous study by Timpson and colleagues (Timpson et al., 2005), our research did not find a significant association between the *CRP* gene and the metabolic syndrome. However, we showed an interaction between *CRP* rs1205 and affective status on the risk of the metabolic syndrome. Our finding of adolescent emotional problems being associated with elevated risk for the metabolic syndrome only in rs1205 CC homozygotes may be linked to their higher CRP levels. According the study by Halder et al., C allele carriers had a higher mean CRP level than the TT genotype (Halder et al., 2010). Consistently with this finding, we showed that depressive symptoms were associated with higher risk of the metabolic syndrome only in CC homozygotes, possibly through higher level of inflammation. The same study also reported interaction effect between three-marker haplotype (A–G–T, rs1417938–rs1800947–rs1205) and depressive symptoms on the higher level of CRP (Halder et al., 2010). Unfortunately, our results are not directly comparable with these findings, since we do not have the information on the two other SNPs. It is possible that this three-marker haplotype, with T allele of rs1205, captures another functional significant variant within *CRP* gene.

Our findings are in line with the following hypothesis explaining the association between depression and the metabolic syndrome: that depression dysregulates immune system pathways in ways that promote inflammation and through inflammation lead to higher risk of the metabolic syndrome. Recent studies have shown that early life trauma, with or without clinical depression, is associated with clinically significant levels of inflammation in adulthood (Danese et al., 2007; Pace et al., 2006). Stress system activation might promote inflammation process through several mechanisms: through activation of the sympathetic nervous system, through vagal withdrawal or through the development of glucocorticoid resistance associated with increased cytokine production (Raison et al., 2006). Thus, HPA axis hyperactivity and autonomic nervous system dysfunction could be one plausible mechanism that explains how emotional problems in adolescence affect the metabolic syndrome in adulthood via the inflammation process (Kop and Gottdiener, 2005).

In conclusion, we find that adolescent emotional problems are associated with the metabolic syndrome 40 years later, in women but not in men, although this sex difference was not statistically significant. We also show that a *CRP* polymorphism modifies the association between adolescent affective status and the metabolic syndrome. This suggests that inflammatory system genes could provide a link between depression and the metabolic syndrome but through more complex interactions than simple associations.

## Conflict of interest statement

Funding organisations had no role in design and conduct of the study or in preparation of the manuscript. The authors have no conflict of interests to disclosure.

## Figures and Tables

**Table 1:** Numbers (%) of study participants with the metabolic syndrome or its components at age 53 by presence or absence of emotional problems in adolescence and affective symptoms in adulthood in total sample.

	Controls, *N* (%)	Cases, *N* (%)	Difference in prevalence^a^ (95% CI)	OR (95% CI)
*Adolescence emotional problems*				
Metabolic syndrome	1841 (28.2)	237 (32.9)	4.7 (−1.6, 11.1)	1.30 (0.98, 1.74)
High waist circumference	2333 (33.9)	307 (35.7)	1.8 (−3.9, 7.5)	1.06 (0.82, 1.36)
Hypertension	2293 (68.5)	300 (69.0)	0.5 (−5.1, 6.0)	1.11 (0.85, 1.44)
High HbA1c level	2046 (26.0)	263 (33.4)	7.7 (1.7, 13.7)	1.46 (1.11, 1.93)
High triglycerides level	2032 (52.5)	260 (52.3)	0.2 (−6.6, 6.3)	1.10 (0.84, 1.43)
High HDL level	1889 (10.3)	245 (9.4)	−1.0 (−4.8, 3.0)	0.90 (0.57, 1.42)

*Adult affective symptoms*				
Metabolic syndrome	1973 (28.4)	132 (34.1)	5.7 (−2.6, 14.0)	1.41 (0.97, 2.05)
High waist circumference	2496 (34.0)	163 (40.5)	6.5 (−1.3, 14.3)	1.26 (0.91, 1.74)
Hypertension	2460 (68.4)	154 (69.4)	1.0 (−6.5, 8.4)	1.22 (0.85, 1.74)
High HbA1c level	2186 (27.1)	144 (27.6)	0.5 (−7.0, 8.1)	1.04 (0.71, 1.51)
High triglycerides level	2163 (52.2)	149 (55.7)	3.5 (−4.7, 11.8)	1.40 (0.99, 1.98)
High HDL level	2019 (10.1)	140 (10.7)	0.7 (−4.6, 6.0)	1.09 (0.63, 1.91)

a Difference in prevalence of metabolic syndrome and its components between those with and without adolescent emotional problems/adult affective symptoms, %.

**Table 2:** Associations between affective status in adolescence and adulthood, and the metabolic syndrome and its components.

	Men				Women			
	Controls, *N* (%)	Cases, *N* (%)	Difference in prevalence^a^ (95% CI)	OR (95% CI)	Controls, *N* (%)	Cases, *N* (%)	Difference in prevalence (95% CI)	OR (95% CI)
*Adolescent emotional problems*								
Metabolic syndrome	906 (33.3)	98 (34.7)	1.4 (−8.6, 11.3)	1.06 (0.69, 1.65)	935 (23.2)	139 (31.7)	8.5 (0.03, 16.6)	1.53 (1.04, 2.26)
High waist circumference	1180 (31.7)	121 (30.3)	−1.3 (−10.0, 7.2)	0.94 (0.63, 1.41)	1152 (36.2)	186 (39.3)	3.1 (−4.5, 10.6)	1.13 (0.83, 1.56)
Hypertension	1170 (76.5)	121 (75.4)	−1.1 (−9.1, 6.9)	0.94 (0.61, 1.45)	1123 (60.2)	179 (64.6)	4.4 (−3.1, 12.0)	1.21 (0.87, 1.68)
High HbA1c level	1050 (26.8)	109 (36.7)	9.9 (0.5, 19.3)	1.58 (1.05, 2.39)	996 (25.1)	154 (31.6)	6.5 (−1.3, 14.3)	1.38 (0.95, 1.99)
High triglyceride level	1039 (64.7)	108 (60.2)	−4.5 (−14.2, 5.2)	0.83 (0.55, 1.24)	993 (39.7)	152 (46.7)	7.0 (−1.5, 15.5)	1.33 (0.95, 1.88)
Low HDL cholesterol level	927 (10.5)	99 (9.1)	−1.4 (−7.4, 4.6)	0.86 (0.42, 1.75)	962 (10.2)	146 (9.6)	−0.6 (−5.7, 4.6)	0.94 (0.52, 1.69)

*Adult affective symptoms*								
Metabolic syndrome	967 (33.1)	43 (37.2)	4.1 (−10.6, 18.9)	1.20 (0.64, 2.26)	1006 (23.9)	89 (32.6)	8.7 (−1.4, 18.8)	1.54 (0.97, 2.46)
High waist circumference	1244 (31.3)	48 (31.3)	0.0 (−13.4, 13.3)	0.99 (0.54, 1.86)	1252 (36.7)	115 (44.4)	7.8 (−1.8, 17.2)	1.38 (0.94, 2.02)
Hypertension	1235 (76.2)	47 (89.4)	13.2 (4.0, 22.3)	2.62 (1.03, 6.69)	1225 (60.7)	107 (61.0)	0.3 (−9.3, 9.8)	1.01 (0.68, 1.51)
High HbA1c level	1108 (27.2)	45 (22.2)	−5.5 (−17.9, 6.9)	0.75 (0.36, 1.52)	1078 (26.4)	99 (30.0)	3.6 (−5.7, 13.0)	1.20 (0.76, 1.87)
High triglyceride level	1094 (63.5)	46 (71.7)	8.2 (−5.1, 21.5)	1.46 (0.76, 2.80)	1069 (40.6)	103 (48.5)	7.9 (−2.2, 18.0)	1.38 (0.92, 2.07)
Low HDL cholesterol level	984 (10.7)	45 (8.9)	−1.8 (−10.3, 6.8)	0.82 (0.29, 2.33)	1035 (9.5)	95 (11.6)	2.1 (−4.6, 8.8)	1.25 (0.65, 2.43)

a Difference in prevalence of metabolic syndrome and its components between those with and without adolescent emotional problems/adult affective symptoms,%.

**Table 3:** Associations between *CRP* gene polymorphisms and metabolic syndrome, and affective status in adolescence and adulthood: genotype and allele frequencies presented by categories of presence of metabolic syndrome, adolescent emotional problems and adult affective symptoms.

	rs1205 genotype distribution, *N* (%)				rs3093068 genotype distribution, *N* (%)			
	N	CC	CT	TT	N	CC	CG	GG
*Metabolic syndrome*								
No	1482	668 (0.45)	670 (0.45)	144 (0.10)	1477	1312 (0.89)	161 (0.11)	4 (0.00)
Yes	618	272 (0.44)	282 (0.46)	64 (0.10)	616	545 (0.88)	70 (0.11)	1 (0.01)
*p* value (genotype model)		0.86				0.89		
*p* value (allele model)		0.59				0.88		

*Adolescent emotional problems*								
No	1976	878 (0.44)	897 (0.45)	201 (0.11)	1971	1757 (0.89)	208 (0.10)	6 (0.01)
Yes	245	107 (0.44)	111 (0.45)	27 (0.11)	243	214 (0.88)	29 (0.12)	0 (0.00)
*p* value (genotype model)		0.91				0.56		
*p* value (allele model)		0.72				0.73		

*Adult affective symptoms*								
No	2087	922 (0.44)	948 (0.45)	217 (0.11)	2081	1860 (0.89)	216 (0.10)	5 (0.01)
Yes	144	69 (0.48)	63 (0.44)	12 (0.08)	144	124 (0.86)	20 (0.14)	0 (0.00)
*p* value (genotype model)		0.59				0.36		
*p* value (allele model)		0.31				0.28		
